# Bis[1-meth­oxy-2,2,2-tris­(pyrazol-1-yl-κ*N* ^2^)ethane]­nickel(II) bis­(tri­fluoro­methane­sulfonate) dihydrate

**DOI:** 10.1107/S1600536813024252

**Published:** 2013-09-04

**Authors:** Ganna Lyubartseva, Sean Parkin, Uma Prasad Mallik

**Affiliations:** a Department of Biochemistry, Chemistry and Physics, Southern Arkansas University, Magnolia, AR 71753, USA; b Department of Chemistry, University of Kentucky, Lexington, KY 40506, USA

## Abstract

In the title salt, [Ni(C_12_H_14_N_6_O)_2_](CF_3_SO_3_)_2_·2H_2_O, the Ni^II^ cation is located on an inversion centre and is coordinated by six N atoms from two tridentate 1-meth­oxy-2,2,2-tris­(pyrazol-1-yl)ethane ligands in a distorted octa­hedral geometry. The Ni—N distances range from 2.0594 (12) to 2.0664 (12) Å, intra-ligand N—Ni—N angles range from 84.59 (5) to 86.06 (5)°, and adjacent inter-ligand N—Ni—N angles range between 93.94 (5) and 95.41 (5)°. In the crystal, inversion-related pyrazole rings are π–π stacked, with an inter­planar spacing of 3.4494 (18) Å, forming chains that propagate parallel to the *a-*axis direction. Inter­molecular O—H⋯O hydrogen bonds are present between water mol­ecules and tri­fluoro­methane­sulfonate anions.

## Related literature

Pyrazole-based tridentate ligands are drawing more attention because of their topology and nature of donor atoms, see: Paulo *et al.* (2004 ▶); Bigmore *et al.* (2005 ▶). For the ligand synthesis, see: Maria *et al.* (2007 ▶). The compound reported here was prepared as part of our ongoing research effort to study nitro­gen-donor tridentate scorpionate ligands coordinated to nickel, see: Lyubartseva *et al.* (2011 ▶, 2012 ▶); Lyubartseva & Parkin (2009 ▶).
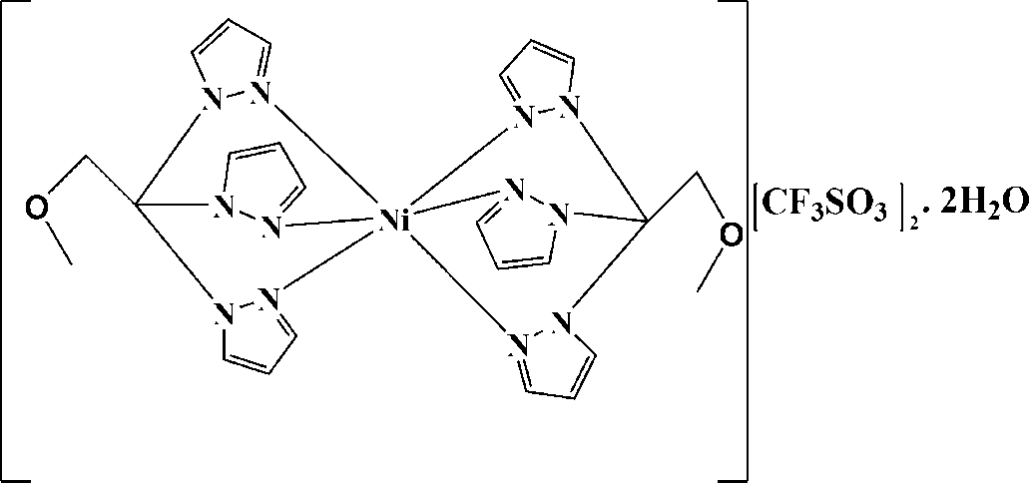


## Experimental

### 

#### Crystal data

[Ni(C_12_H_14_N_6_O)_2_](CF_3_O_3_S)_2_·2H_2_O
*M* *_r_* = 909.46
Triclinic, 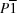

*a* = 8.5582 (2) Å
*b* = 9.6515 (2) Å
*c* = 12.2347 (2) Å
α = 110.399 (1)°
β = 103.665 (1)°
γ = 97.317 (1)°
*V* = 895.66 (3) Å^3^
*Z* = 1
Mo *K*α radiation
μ = 0.76 mm^−1^
*T* = 90 K
0.26 × 0.22 × 0.15 mm

#### Data collection

Nonius KappaCCD diffractometer
Absorption correction: multi-scan (*SADABS*; Sheldrick, 2008*a* ▶) *T*_min_ = 0.760, *T*_max_ = 0.862
25573 measured reflections
4095 independent reflections
3708 reflections with *I* > 2σ(*I*)
*R*_int_ = 0.023

#### Refinement

*R*[*F* ^2^ > 2σ(*F* ^2^)] = 0.029
*wR*(*F* ^2^) = 0.072
*S* = 1.07
4095 reflections
266 parameters
H atoms treated by a mixture of independent and constrained refinement
Δρ_max_ = 0.47 e Å^−3^
Δρ_min_ = −0.49 e Å^−3^

### 

Data collection: *COLLECT* (Nonius, 1998 ▶); cell refinement: *SCALEPACK* (Otwinowski & Minor, 1997 ▶); data reduction: *DENZO-SMN* (Otwinowski & Minor, 1997 ▶); program(s) used to solve structure: *SHELXS97* (Sheldrick, 2008*b* ▶); program(s) used to refine structure: *SHELXL2013* (Sheldrick, 2008*b* ▶); molecular graphics: *XP* in *SHELXTL* (Sheldrick, 2008*b* ▶); software used to prepare material for publication: *SHELXL2013*.

## Supplementary Material

Crystal structure: contains datablock(s) global, I. DOI: 10.1107/S1600536813024252/tk5252sup1.cif

Structure factors: contains datablock(s) I. DOI: 10.1107/S1600536813024252/tk5252Isup2.hkl

Additional supplementary materials:  crystallographic information; 3D view; checkCIF report

## Figures and Tables

**Table 1:** Hydrogen-bond geometry (Å, °)

*D*—H⋯*A*	*D*—H	H⋯*A*	*D*⋯*A*	*D*—H⋯*A*
O1*W*—H2*W*⋯O3*A*	0.94 (3)	2.06 (3)	2.994 (2)	174 (2)
O1*W*—H1*W*⋯O3*A* ^i^	0.97 (3)	2.12 (3)	3.0613 (19)	163 (2)

Symmetry code: (i) 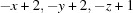.

**Fig. 1.**
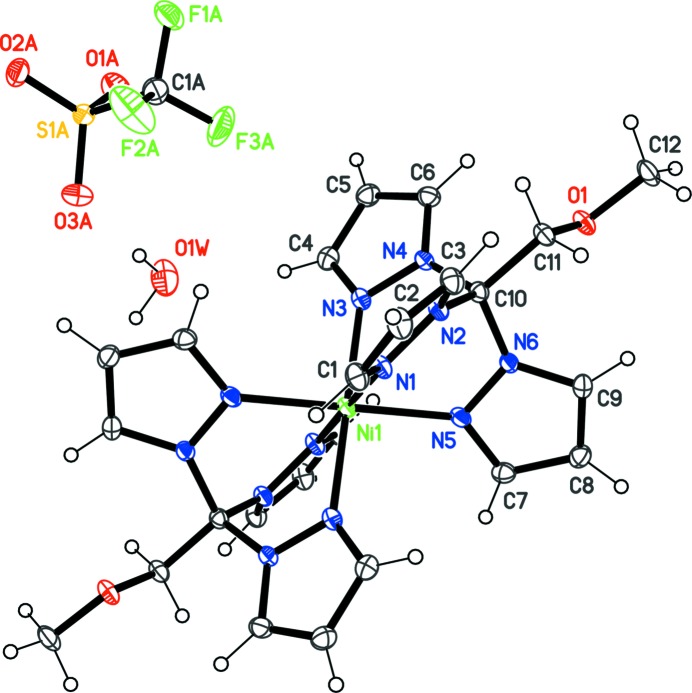
View of the title compound with displacement ellipsoids drawn at the 50% probability level. Unlabelled atoms are related to their labelled counterparts by inversion (1/2 - x, 1.5 - y, 1 - z).

## supplementary crystallographic information

### 1. Comment

The described structure was studied in continuation of on-going studies (Lyubartseva & Parkin, 2009; Lyubartseva *et al.*, 2011, 2012) owing to interest in pyrazole-based tridentate ligands (Paulo *et al.*, 2004; Bigmore *et al.*, 2005). In an attempt to prepare mononuclear [LNi^II^(CN)_3_]^-^, where *L* is 1-methoxy-2,2,2-tris(pyrazol-1-yl)ethane, a tridentate neutral nitrogen donor ligand, we isolated the major product [Ni(C_12_H_14_N_6_0_2_)_2_][CF_3_SO_3_]_2_·2H_2_O as light-pink triclinic crystals. In the crystal, the Ni(II) cation is situated on an inversion centre and is coordinated by six N atoms from the two tridentate tpmOMe ligands, Fig. 1, (average Ni—N distance = 2.062 Å) in a distorted octahedral geometry. The average N—Ni—N angle between adjacent pyrazole-ring coordinated N atoms is 85.13° for the six acute angles and 94.87° for the six obtuse angles. In the crystal, inversion-related (-*x*, 1 - *y*, 1 - *z*) pyrazole rings are π—π stacked, with an interplanar spacing of 3.4494 (18) Å, forming chains that propagate parallel to the *a* axis. Intramolecular O—H···O hydrogen bonds are present between water and trifluoromethanesulfonate anion, Table 1.

### 2. Experimental

1-Methoxy-2,2,2-tris(pyrazol-1-yl)ethane ligand was synthesized according to the previously published procedure of Maria *et al.* (2007). Nickel trifluoromethanesulfonate was used as received. Ni(OTf)_2_ (358 mg, 1 mmol), 1-methoxy-2,2,2-tris(pyrazol-1-yl)ethane (258 mg, 1 mmol) and NEt_4_CN (312 mg, 2 mmol) were suspended in a mixture of methanol (20 ml) and water (10 ml), and stirred for 30 minutes. The resulting solution was filtered and solvent was slowly evaporated in air. Light-pink crystals were obtained after 3 weeks (294 mg, 64.6% yield). Elemental analysis, calculated for C_26_H_32_F_6_N_12_NiO_10_S_2_: C 34.34, H 3.55, N 18.48; found C 34.64, H 3.40, N 18.35. IR (cm^-1^): 3624, 3487, 3145, 2920, 1615, 1523, 1410, 1388, 1340, 1323, 1257, 1225, 1199, 1164, 1105, 1069, 1059, 1028, 1010, 973, 919, 854, 755, 674, 653, 635, 602, 572, 516.

### 3. Refinement

H atoms were found in difference Fourier maps. Water hydrogen atom coordinates were refined freely, but with *U*_iso_(H) values set to 1.5*U*_eq_ O_water_. All other H atoms were placed at idealized positions with constrained distances of 0.98 Å (RCH_3_), 0.99 Å (*R*_2_CH_2_), 0.95 Å (C_sp2_H), and with *U*_iso_(H) values set to either 1.2*U*_eq_ or 1.5*U*_eq_ (RCH_3_) of the attached atom.

### Crystal data

**Table:**

[Ni(C_12_H_14_N_6_O)_2_](CF_3_O_3_S)_2_·2H_2_O	*Z* = 1
*M**_r_* = 909.46	*F*(000) = 466
Triclinic, *P*1	*D*_x_ = 1.686 Mg m^−^^3^
*a* = 8.5582 (2) Å	Mo *K*α radiation, λ = 0.71073 Å
*b* = 9.6515 (2) Å	Cell parameters from 25674 reflections
*c* = 12.2347 (2) Å	θ = 1.0–27.5°
α = 110.399 (1)°	µ = 0.76 mm^−^^1^
β = 103.665 (1)°	*T* = 90 K
γ = 97.317 (1)°	Block, pink
*V* = 895.66 (3) Å^3^	0.26 × 0.22 × 0.15 mm

### Data collection

**Table:**

Nonius KappaCCD diffractometer	4095 independent reflections
Radiation source: fine-focus sealed-tube	3708 reflections with *I* > 2σ(*I*)
Detector resolution: 9.1 pixels mm^-1^	*R*_int_ = 0.023
φ and ω scans at fixed χ = 55°	θ_max_ = 27.5°, θ_min_ = 1.9°
Absorption correction: multi-scan (*SADABS*; Sheldrick, 2008*a*)	*h* = −11→11
*T*_min_ = 0.760, *T*_max_ = 0.862	*k* = −12→12
25573 measured reflections	*l* = −15→15

### Refinement

**Table:**

Refinement on *F*^2^	Primary atom site location: structure-invariant direct methods
Least-squares matrix: full	Secondary atom site location: difference Fourier map
*R*[*F*^2^ > 2σ(*F*^2^)] = 0.029	Hydrogen site location: difference Fourier map
*wR*(*F*^2^) = 0.072	H atoms treated by a mixture of independent and constrained refinement
*S* = 1.07	*w* = 1/[σ^2^(*F*_o_^2^) + (0.0311*P*)^2^ + 0.6676*P*] where *P* = (*F*_o_^2^ + 2*F*_c_^2^)/3
4095 reflections	(Δ/σ)_max_ < 0.001
266 parameters	Δρ_max_ = 0.47 e Å^−^^3^
0 restraints	Δρ_min_ = −0.49 e Å^−^^3^

### Special details

**Table:**

Geometry. All e.s.d.'s (except the e.s.d. in the dihedral angle between two l.s. planes) are estimated using the full covariance matrix. The cell e.s.d.'s are taken into account individually in the estimation of e.s.d.'s in distances, angles and torsion angles; correlations between e.s.d.'s in cell parameters are only used when they are defined by crystal symmetry. An approximate (isotropic) treatment of cell e.s.d.'s is used for estimating e.s.d.'s involving l.s. planes.

### Fractional atomic coordinates and isotropic or equivalent isotropic displacement parameters (Å^2^)

**Table:**

	*x*	*y*	*z*	*U*_iso_*/*U*_eq_	
Ni1	0.5000	0.5000	0.5000	0.01222 (8)	
N1	0.44622 (15)	0.29426 (14)	0.35443 (11)	0.0145 (3)	
N2	0.30215 (15)	0.25366 (14)	0.26196 (11)	0.0130 (2)	
C1	0.52676 (19)	0.18620 (17)	0.31824 (14)	0.0173 (3)	
H1	0.6315	0.1839	0.3649	0.021*	
C2	0.43694 (19)	0.07609 (18)	0.20223 (15)	0.0191 (3)	
H2	0.4683	−0.0117	0.1563	0.023*	
C3	0.29420 (19)	0.12165 (17)	0.16891 (14)	0.0167 (3)	
H3	0.2062	0.0707	0.0948	0.020*	
N3	0.39074 (15)	0.58147 (14)	0.37338 (11)	0.0149 (3)	
N4	0.24408 (15)	0.49654 (14)	0.28723 (11)	0.0130 (2)	
C4	0.4154 (2)	0.71338 (18)	0.36109 (14)	0.0173 (3)	
H4	0.5085	0.7955	0.4093	0.021*	
C5	0.2853 (2)	0.71481 (18)	0.26739 (15)	0.0192 (3)	
H5	0.2738	0.7953	0.2407	0.023*	
C6	0.17837 (19)	0.57573 (18)	0.22245 (14)	0.0169 (3)	
H6	0.0772	0.5411	0.1581	0.020*	
N5	0.26178 (15)	0.43228 (14)	0.50146 (11)	0.0139 (2)	
N6	0.14141 (15)	0.35547 (14)	0.39195 (11)	0.0125 (2)	
C7	0.19545 (19)	0.41516 (17)	0.58503 (14)	0.0160 (3)	
H7	0.2508	0.4571	0.6704	0.019*	
C8	0.03292 (19)	0.32692 (18)	0.53068 (14)	0.0170 (3)	
H8	−0.0406	0.2992	0.5708	0.020*	
C9	0.00275 (18)	0.28935 (17)	0.40787 (14)	0.0154 (3)	
H9	−0.0963	0.2285	0.3453	0.018*	
C10	0.17782 (18)	0.34450 (16)	0.27853 (13)	0.0127 (3)	
C11	0.02317 (18)	0.26347 (17)	0.16833 (13)	0.0147 (3)	
H11A	0.0485	0.2605	0.0928	0.018*	
H11B	−0.0137	0.1577	0.1596	0.018*	
O1	−0.10362 (13)	0.34191 (12)	0.18516 (10)	0.0167 (2)	
C12	−0.25257 (19)	0.26218 (19)	0.08624 (15)	0.0204 (3)	
H12A	−0.2344	0.2595	0.0095	0.031*	
H12B	−0.3413	0.3143	0.1009	0.031*	
H12C	−0.2839	0.1581	0.0807	0.031*	
S1A	0.86275 (5)	0.86391 (4)	0.17962 (3)	0.01557 (9)	
O1A	0.76221 (16)	0.97224 (14)	0.17899 (12)	0.0287 (3)	
O2A	0.98159 (15)	0.86458 (15)	0.11398 (11)	0.0272 (3)	
O3A	0.92445 (16)	0.85421 (15)	0.29641 (11)	0.0269 (3)	
F1A	0.64625 (13)	0.67273 (12)	−0.02656 (9)	0.0292 (2)	
F2A	0.79191 (16)	0.56755 (13)	0.07329 (13)	0.0497 (4)	
F3A	0.59605 (16)	0.65956 (16)	0.13326 (12)	0.0500 (4)	
C1A	0.7161 (2)	0.68201 (19)	0.08608 (16)	0.0220 (3)	
O1W	0.76122 (18)	0.99331 (17)	0.48585 (14)	0.0368 (3)	
H1W	0.848 (3)	1.032 (3)	0.563 (3)	0.055*	
H2W	0.812 (3)	0.944 (3)	0.429 (3)	0.055*	

### Atomic displacement parameters (Å^2^)

**Table:**

	*U*^11^	*U*^22^	*U*^33^	*U*^12^	*U*^13^	*U*^23^
Ni1	0.01029 (13)	0.01323 (13)	0.01154 (13)	0.00243 (10)	0.00191 (10)	0.00400 (10)
N1	0.0101 (6)	0.0164 (6)	0.0136 (6)	0.0031 (5)	0.0010 (5)	0.0037 (5)
N2	0.0102 (6)	0.0146 (6)	0.0119 (6)	0.0027 (5)	0.0019 (5)	0.0037 (5)
C1	0.0134 (7)	0.0180 (7)	0.0198 (8)	0.0051 (6)	0.0045 (6)	0.0062 (6)
C2	0.0162 (8)	0.0165 (7)	0.0214 (8)	0.0050 (6)	0.0065 (6)	0.0029 (6)
C3	0.0150 (7)	0.0159 (7)	0.0152 (7)	0.0021 (6)	0.0040 (6)	0.0022 (6)
N3	0.0117 (6)	0.0157 (6)	0.0142 (6)	0.0005 (5)	0.0012 (5)	0.0050 (5)
N4	0.0105 (6)	0.0148 (6)	0.0117 (6)	0.0019 (5)	0.0011 (5)	0.0048 (5)
C4	0.0173 (7)	0.0164 (7)	0.0186 (7)	0.0025 (6)	0.0063 (6)	0.0070 (6)
C5	0.0202 (8)	0.0193 (7)	0.0218 (8)	0.0052 (6)	0.0068 (6)	0.0119 (6)
C6	0.0160 (7)	0.0210 (7)	0.0159 (7)	0.0065 (6)	0.0043 (6)	0.0095 (6)
N5	0.0119 (6)	0.0166 (6)	0.0105 (6)	0.0030 (5)	0.0014 (5)	0.0036 (5)
N6	0.0104 (6)	0.0146 (6)	0.0106 (6)	0.0017 (5)	0.0021 (5)	0.0038 (5)
C7	0.0177 (7)	0.0181 (7)	0.0132 (7)	0.0058 (6)	0.0054 (6)	0.0061 (6)
C8	0.0164 (7)	0.0191 (7)	0.0189 (8)	0.0047 (6)	0.0081 (6)	0.0093 (6)
C9	0.0120 (7)	0.0151 (7)	0.0189 (7)	0.0023 (5)	0.0049 (6)	0.0066 (6)
C10	0.0113 (7)	0.0141 (7)	0.0127 (7)	0.0036 (5)	0.0039 (5)	0.0047 (5)
C11	0.0113 (7)	0.0170 (7)	0.0124 (7)	0.0032 (6)	0.0013 (6)	0.0032 (6)
O1	0.0108 (5)	0.0193 (5)	0.0157 (5)	0.0048 (4)	0.0002 (4)	0.0039 (4)
C12	0.0122 (7)	0.0224 (8)	0.0213 (8)	0.0019 (6)	−0.0030 (6)	0.0082 (7)
S1A	0.01472 (18)	0.01723 (18)	0.01564 (18)	0.00405 (14)	0.00441 (14)	0.00749 (14)
O1A	0.0262 (7)	0.0202 (6)	0.0351 (7)	0.0104 (5)	0.0029 (5)	0.0081 (5)
O2A	0.0200 (6)	0.0363 (7)	0.0205 (6)	−0.0021 (5)	0.0088 (5)	0.0067 (5)
O3A	0.0297 (7)	0.0335 (7)	0.0178 (6)	0.0073 (5)	0.0035 (5)	0.0128 (5)
F1A	0.0236 (5)	0.0295 (5)	0.0251 (5)	−0.0015 (4)	−0.0035 (4)	0.0091 (4)
F2A	0.0461 (8)	0.0180 (5)	0.0645 (9)	0.0101 (5)	−0.0122 (6)	0.0102 (6)
F3A	0.0399 (7)	0.0563 (8)	0.0458 (7)	−0.0180 (6)	0.0195 (6)	0.0166 (6)
C1A	0.0208 (8)	0.0204 (8)	0.0266 (9)	0.0036 (6)	0.0052 (7)	0.0129 (7)
O1W	0.0266 (7)	0.0383 (8)	0.0339 (8)	0.0027 (6)	0.0103 (6)	0.0014 (6)

### Geometric parameters (Å, º)

**Table:**

Ni1—N1	2.0594 (12)	N5—N6	1.3664 (17)
Ni1—N1^i^	2.0594 (12)	N6—C9	1.3625 (19)
Ni1—N3^i^	2.0602 (13)	N6—C10	1.4643 (18)
Ni1—N3	2.0602 (13)	C7—C8	1.403 (2)
Ni1—N5	2.0664 (12)	C7—H7	0.9500
Ni1—N5^i^	2.0664 (12)	C8—C9	1.367 (2)
N1—C1	1.3283 (19)	C8—H8	0.9500
N1—N2	1.3648 (17)	C9—H9	0.9500
N2—C3	1.3617 (19)	C10—C11	1.529 (2)
N2—C10	1.4689 (18)	C11—O1	1.4140 (18)
C1—C2	1.400 (2)	C11—H11A	0.9900
C1—H1	0.9500	C11—H11B	0.9900
C2—C3	1.370 (2)	O1—C12	1.4331 (18)
C2—H2	0.9500	C12—H12A	0.9800
C3—H3	0.9500	C12—H12B	0.9800
N3—C4	1.330 (2)	C12—H12C	0.9800
N3—N4	1.3671 (17)	S1A—O1A	1.4371 (12)
N4—C6	1.3600 (19)	S1A—O2A	1.4378 (12)
N4—C10	1.4618 (18)	S1A—O3A	1.4418 (12)
C4—C5	1.402 (2)	S1A—C1A	1.8237 (17)
C4—H4	0.9500	F1A—C1A	1.333 (2)
C5—C6	1.370 (2)	F2A—C1A	1.332 (2)
C5—H5	0.9500	F3A—C1A	1.322 (2)
C6—H6	0.9500	O1W—H1W	0.97 (3)
N5—C7	1.3278 (19)	O1W—H2W	0.94 (3)
			
N1—Ni1—N1^i^	180.0	C7—N5—Ni1	134.47 (10)
N1—Ni1—N3^i^	93.94 (5)	N6—N5—Ni1	118.35 (9)
N1^i^—Ni1—N3^i^	86.06 (5)	C9—N6—N5	110.86 (12)
N1—Ni1—N3	86.06 (5)	C9—N6—C10	129.46 (12)
N1^i^—Ni1—N3	93.94 (5)	N5—N6—C10	119.50 (12)
N3^i^—Ni1—N3	180.0	N5—C7—C8	111.13 (14)
N1—Ni1—N5	84.59 (5)	N5—C7—H7	124.4
N1^i^—Ni1—N5	95.41 (5)	C8—C7—H7	124.4
N3^i^—Ni1—N5	95.27 (5)	C9—C8—C7	105.44 (13)
N3—Ni1—N5	84.73 (5)	C9—C8—H8	127.3
N1—Ni1—N5^i^	95.41 (5)	C7—C8—H8	127.3
N1^i^—Ni1—N5^i^	84.59 (5)	N6—C9—C8	107.16 (13)
N3^i^—Ni1—N5^i^	84.73 (5)	N6—C9—H9	126.4
N3—Ni1—N5^i^	95.27 (5)	C8—C9—H9	126.4
N5—Ni1—N5^i^	180.00 (7)	N4—C10—N6	109.51 (11)
C1—N1—N2	105.55 (12)	N4—C10—N2	109.13 (11)
C1—N1—Ni1	135.27 (11)	N6—C10—N2	108.63 (11)
N2—N1—Ni1	118.89 (9)	N4—C10—C11	111.11 (12)
C3—N2—N1	110.80 (12)	N6—C10—C11	110.71 (12)
C3—N2—C10	129.99 (12)	N2—C10—C11	107.69 (11)
N1—N2—C10	118.99 (11)	O1—C11—C10	109.35 (12)
N1—C1—C2	111.08 (14)	O1—C11—H11A	109.8
N1—C1—H1	124.5	C10—C11—H11A	109.8
C2—C1—H1	124.5	O1—C11—H11B	109.8
C3—C2—C1	105.45 (14)	C10—C11—H11B	109.8
C3—C2—H2	127.3	H11A—C11—H11B	108.3
C1—C2—H2	127.3	C11—O1—C12	110.04 (11)
N2—C3—C2	107.11 (13)	O1—C12—H12A	109.5
N2—C3—H3	126.4	O1—C12—H12B	109.5
C2—C3—H3	126.4	H12A—C12—H12B	109.5
C4—N3—N4	105.69 (12)	O1—C12—H12C	109.5
C4—N3—Ni1	135.09 (11)	H12A—C12—H12C	109.5
N4—N3—Ni1	118.51 (9)	H12B—C12—H12C	109.5
C6—N4—N3	110.71 (12)	O1A—S1A—O2A	114.77 (8)
C6—N4—C10	129.53 (13)	O1A—S1A—O3A	114.93 (8)
N3—N4—C10	119.72 (12)	O2A—S1A—O3A	114.87 (8)
N3—C4—C5	110.81 (14)	O1A—S1A—C1A	103.12 (8)
N3—C4—H4	124.6	O2A—S1A—C1A	103.04 (8)
C5—C4—H4	124.6	O3A—S1A—C1A	103.80 (8)
C6—C5—C4	105.58 (14)	F3A—C1A—F2A	108.54 (15)
C6—C5—H5	127.2	F3A—C1A—F1A	107.47 (14)
C4—C5—H5	127.2	F2A—C1A—F1A	106.24 (14)
N4—C6—C5	107.21 (14)	F3A—C1A—S1A	112.04 (12)
N4—C6—H6	126.4	F2A—C1A—S1A	110.88 (12)
C5—C6—H6	126.4	F1A—C1A—S1A	111.42 (11)
C7—N5—N6	105.40 (12)	H1W—O1W—H2W	104 (2)
			
C1—N1—N2—C3	−0.31 (16)	N3—N4—C10—N6	63.30 (16)
Ni1—N1—N2—C3	174.45 (10)	C6—N4—C10—N2	126.87 (15)
C1—N1—N2—C10	174.92 (12)	N3—N4—C10—N2	−55.48 (16)
Ni1—N1—N2—C10	−10.32 (16)	C6—N4—C10—C11	8.3 (2)
N2—N1—C1—C2	0.58 (17)	N3—N4—C10—C11	−174.09 (12)
Ni1—N1—C1—C2	−172.90 (11)	C9—N6—C10—N4	132.03 (15)
N1—C1—C2—C3	−0.63 (19)	N5—N6—C10—N4	−53.32 (16)
N1—N2—C3—C2	−0.07 (17)	C9—N6—C10—N2	−108.88 (16)
C10—N2—C3—C2	−174.63 (14)	N5—N6—C10—N2	65.78 (16)
C1—C2—C3—N2	0.41 (18)	C9—N6—C10—C11	9.2 (2)
C4—N3—N4—C6	−0.20 (16)	N5—N6—C10—C11	−176.17 (12)
Ni1—N3—N4—C6	171.53 (10)	C3—N2—C10—N4	−120.06 (16)
C4—N3—N4—C10	−178.26 (12)	N1—N2—C10—N4	65.75 (16)
Ni1—N3—N4—C10	−6.53 (16)	C3—N2—C10—N6	120.60 (16)
N4—N3—C4—C5	0.07 (17)	N1—N2—C10—N6	−53.58 (16)
Ni1—N3—C4—C5	−169.62 (11)	C3—N2—C10—C11	0.7 (2)
N3—C4—C5—C6	0.09 (18)	N1—N2—C10—C11	−173.53 (12)
N3—N4—C6—C5	0.26 (17)	N4—C10—C11—O1	−64.66 (15)
C10—N4—C6—C5	178.07 (14)	N6—C10—C11—O1	57.25 (15)
C4—C5—C6—N4	−0.20 (17)	N2—C10—C11—O1	175.87 (11)
C7—N5—N6—C9	−1.01 (16)	C10—C11—O1—C12	−176.45 (12)
Ni1—N5—N6—C9	166.00 (10)	O1A—S1A—C1A—F3A	−60.94 (14)
C7—N5—N6—C10	−176.59 (12)	O2A—S1A—C1A—F3A	179.35 (13)
Ni1—N5—N6—C10	−9.58 (16)	O3A—S1A—C1A—F3A	59.25 (14)
N6—N5—C7—C8	0.35 (17)	O1A—S1A—C1A—F2A	177.62 (13)
Ni1—N5—C7—C8	−163.56 (11)	O2A—S1A—C1A—F2A	57.91 (14)
N5—C7—C8—C9	0.41 (18)	O3A—S1A—C1A—F2A	−62.19 (14)
N5—N6—C9—C8	1.28 (17)	O1A—S1A—C1A—F1A	59.51 (14)
C10—N6—C9—C8	176.30 (14)	O2A—S1A—C1A—F1A	−60.20 (13)
C7—C8—C9—N6	−1.00 (17)	O3A—S1A—C1A—F1A	179.70 (12)
C6—N4—C10—N6	−114.34 (16)		

Symmetry code: (i) −*x*+1, −*y*+1, −*z*+1.

### Hydrogen-bond geometry (Å, º)

**Table:**

*D*—H···*A*	*D*—H	H···*A*	*D*···*A*	*D*—H···*A*
O1*W*—H2*W*···O3*A*	0.94 (3)	2.06 (3)	2.994 (2)	174 (2)
O1*W*—H1*W*···O3*A*^ii^	0.97 (3)	2.12 (3)	3.0613 (19)	163 (2)

Symmetry code: (ii) −*x*+2, −*y*+2, −*z*+1.
